# Newborn screening in mucopolysaccharidoses

**DOI:** 10.1186/s13052-018-0552-3

**Published:** 2018-11-16

**Authors:** Maria Alice Donati, Elisabetta Pasquini, Marco Spada, Giulia Polo, Alberto Burlina

**Affiliations:** 1 Metabolic and Muscular Unit, Regional Reference Centre Expanded Newborn Screening, Meyer Children Hospital, Florence, Italy; 2 Division of Inherited Metabolic Diseases, Regional Center for Expanded Neonatal Screening, Department of Women and Children’s Health, University Hospital of Padova, Via Orus 2/B, 35129 Padova, Italy; 3 Department of Pediatrics, Ospedale Regina Margherita, P.zza Polonia, 94, 10126 Torino, Italy

**Keywords:** Newborn screening, Mucopolysaccharidoses, Mucopolysaccharidosis type I, Lysosomal storage disorders

## Abstract

Newborn screening (NBS) methods and therapeutic options have become increasingly available for mucopolysaccharidoses (MPS), and there is a clear evidence that early intervention significantly improves the outcome. It is recommended that mucopolysaccharidosis type I (MPS I) is included in the US newborn screening panel, and this is currently underway in some NBS programs in the world. The key factors in recommending MPS I for inclusion in NBS are the strongly improved efficacy of early-onset therapy and the improved performance of screening tests. Two studies on MPS I screening have been conducted in Italy. In the Tuscany-Umbria pilot NBS, eight infants were confirmed positive, and alpha-l-iduronidase (IDUA) gene molecular analysis showed that seven had either homozygosity or compound heterozygosity for pseudodeficiency alleles. p.Ala79Thr and p.His82Gln changes were demonstrated in four and three infants, respectively, six of which were of African origin. Only one infant had transitory elevation of urine glycosaminoglycans (GAGs) (by quantitative analysis) and she is in follow-up at the time of writing. In the North East Italy experience, there was one affected newborn for 66,491 screened. In this patient treatment started at 1 month of age. In the North East Italy experience the incidence of pseudodeficiency was very high (1:6044), with a high incidence of pseudodeficiency from patients of African origin.

A significant problem that is encountered in the follow-up of infants with abnormal NBS and variants of unknown significance (VUS) on molecular analysis results relates to those who cannot be positively identified as either affected or unaffected. Long-term follow-up of these infants, and of those detected with late-onset disorders, will be essential to document the true risks and benefits of NBS. The availability of treatments in MPS II, IVA, VI, and VII with a better clinical outcome when started early in life, and the availability of a combined multiple assay for MPS, may be a prerequisite for new pilot NBS studies in the near future.

## Background

Lysosomal storage diseases (LSDs) are inborn errors of metabolism that include 60 different inherited disorders. Together, they have a reported combined incidence of at least one in 1500 to one in 7000 newborns [1]. The current diagnosis facilities for evaluation and the increasing availability of treatment options for LSDs, including enzyme replacement therapy (ERT), hematopoietic stem cell transplantation (HSCT) (alone or in combination with ERT), small molecular weight pharmacologic chaperones, and gene therapy, have stimulated efforts to screen newborns for these disorders.

Among LSDs, mucopolysaccharidoses (MPS) are due to a reduction or lack of lysosomal enzymes with progressive storage of metabolic precursors (glycosaminoglycans (GAGs)) within the lysosomes, resulting in cellular dysfunction and multiple organ damage. Recently, many MPS have been recognized as diseases that could greatly benefit from an early diagnosis because the availability of treatments produces a better clinical outcome when started early in life.

The recent introduction of tandem mass spectrometry (MS/MS) methods to analyze proteins and substrates in dried blood spots (DBS) has facilitated and expanded the diseases detected in newborn screening (NBS) programs [2]. Currently, many programs in developed countries have used the MS/MS technology to detect newborns with 40 to 50 severe metabolic diseases. Pilot LSD screening programs have been implemented in a number of countries worldwide [3–7]. Such programs have shown that NBS for LSDs is feasible and economically justifiable for extension to large populations. Nevertheless, the choice of disease panels for expanded NBS (ENBS) is still controversial, as reflected in the different disease panels in screening programs around the world. Recently, the Recommended Uniform Screening Panel (RUSP) of the US Department of Health and Human Services Secretary’s Advisory Committee on Heritable Disorders in Newborns and Children includes Pompe and MPS I diseases in the primary panel for neonatal screening programs [8].

This paper will review the status of MPS type I newborn screening and present the experience of two Italian centers in this field.

### Methods for detecting MPS in DBS

The diagnosis of MPS is based on the identification of the deficient enzyme activity, usually in leucocytes and/or fibroblasts, and on molecular analysis. Chamoles and coworkers were the first to show that several lysosomal enzymatic activities could be assayed after rehydration of dried blood spot punches with aqueous buffers. They first reported alpha-l-iduronidase (IDUA) activity determination by fluorimetric assay on DBS on filter paper [9]. This matrix provides important advantages in sample handling, storage, and transportation when compared with traditional methods in leukocytes.

Conventional fluorimetric methods have been developed for 14 different lysosomal enzymes. The major limitation of the fluorimetric assays is that this method has restricted capacity for multiplex tests, and only one or two enzymes can be assayed reliably using conventional 96-well microplate technology. The development of digital microfluidic chip technology has converted the conventional fluorimetric enzyme assays into high-throughput methods able to simultaneously assay multiple enzymes [10].

In the past 10 years the use of MS/MS for quantification of lysosomal enzyme activity in DBS has been developed with single and multiplex MS/MS methods [11–13].

MS/MS combines high selectivity and high sensitivity with multiplexing capability. The use of MS/MS has advantages over fluorimetric or spectrophotometric assays in the simultaneous quantification of several markers.

Many of the lysosomal enzyme activity assays can be “multiplexed” by incubating samples with a cocktail containing substrates and internal standards in a common buffer; MS/MS measures products and calculates enzyme activities [14–16]. The robustness of the MS/MS method for multiplex assay of alpha-galactosidase (Fabry disease), acid-alpha-glucosidase (Pompe disease), and IDUA (MPS I) has been evaluated on anonymous newborn DBS [17].

A novel flow-injection MS/MS method using a simple liquid-liquid extraction step has been recently made available to the market (NeoLSD®, Perkin Elmer). This method uses a universal substrate/internal standard reaction mix for the six-plex assay (Pompe, MPS I, Fabry, Gaucher, Niemann Pick A/B, and Krabbe disease) [18]. Recently a study for the identification of newborns at risk of developing Pompe, MPS I, Fabry, Gaucher, Niemann Pick A/B, and Krabbe disease has been performed in the Washington state NBS laboratory [19].

Numerous studies have contributed to the standardization and optimization of the methods, and the development and evaluation of quality control for DBS have contributed to the safety of the results [20–22].

La Marca et al. [23] developed a new method for determining alpha-galactosidase (GLA), acid-alpha-glucosidase (GAA), glucocerebrosidase (ABG), galactocerebrosidase (GALC), and sphingomyelinase (ASM) in MS/MS. This method is simple, cheap, easy to perform, and applicable to a mass screening. This method takes only 4 min as an analysis run-time and without any purification following the enzymatic reaction [23]. In 2013, IDUA activity determination was added to other five enzyme activities for the simultaneous detection of six LSDs [24].

A recent study has assessed the biochemical parameters of the enzymes IDUA and arylsulfatase B (ASB) in DBS on filter paper and, moreover, showed that the stability (at 21 days) of IDUA activity was lower than ASB (at 60 days) [25].

With the development of therapies for several MPS, detection of enzyme activity in DBS from newborns is of interest since the effectiveness of treatment is maximized at the early stage of the disease. Several assays for lysosomal enzymes were developed in a multiplex format using MS/MS combined with liquid chromatography (LC-MS/MS) [18]; a combined multiplex assay for MPS has been reported for IDUA (MPS I), iduronate-2-sulfatase (I2S; MPS II), *N*-acetylgalactosamine-6-sulfatase (GALNS; MPS IV), and *N*-acetylgalactosamine-4-sulfatase (ARSB, MPS VI) [26]. A new multiplex assay expanded the use of LC-MS/MS for multiplex newborn screening of seven lysosomal enzymes in DBS; this new seven-multiplex assay is for enzymes responsible for the mucopolysaccharidoses I, II, IIIB, IVA, VI, and VII and for type 2 neuronal ceroid lipofuscinosis (LICL) [27].

Recently, DBS-based assays measuring GAGs have been developed and proposed for use in NBS to detect MPS I and other MPS [28, 29]. As an alternative to direct measurement of lysosomal enzymes in DBS, MS/MS analysis of accumulated glycosaminoglycans (measured as disaccharide degradation products) has been suggested as a potentially valuable tool for first-tier newborn screening of MPS, especially when considering that a single test can screen several distinct MPS [30]. Measuring GAG levels in DBS is useful for diagnosis and potentially for monitoring the therapeutic efficacy in MPS [31].

### Status of newborn screening for MPS type I

Over the years, MPS I neonatal screening has been conducted for evaluation for inclusion in primary screening programs.

In Taiwan, an NBS pilot program has been conducted for MPS I from 1 October 2008 to 30 April 2013, with 35,286 newborns screened using a fluorimetric assay. Two newborns were recalled and had confirmed deficient leukocyte IDUA activity, and molecular analysis confirmed the diagnosis of MPS I; the incidence in Taiwan estimated from this study is about 1/17,643 [32].

Missouri has been the first US state to screen all newborns for multiple LSDs using a comprehensive population pilot screening study with follow-up care for each disease. For NBS, a full-population pilot study using a multiplexing digital microfluidic fluorimetric enzymatic assay to detect Pompe disease, Fabry disease, Gaucher disease, and MPS I started in 2013 [33, 34]. In the first 6 months of the Missouri LSD pilot study, 43,701 newborns were screened. The authors reported 27 newborns with confirmed screening diagnosis of an LSD genotype, three newborns were confirmed with an MPS I genotype (1 newborn with confirmed disorder and two newborns were classified with a genetic condition of unknown significance), seven newborns with pseudodeficiency, two carriers, and 16 with false-positive results [33]. The incidence rate of 1:14,567 for MPS I is in the same range reported in a previous Taiwanese pilot study (1:17,643) [32]. The NBS pilot study of MPS I in Taiwan with 35,286 newborns founded no pseudodeficiencies, despite DNA sequencing [32].

Another important experience in an NBS program for LSD including MPS I has been reported in Illinois, USA [35]. MS/MS was used to assay for the five LSD-associated enzymes to detect MPS I, Pompe disease, Fabry disease, Gaucher disease, and Niemann-Pick disease type A/B in DBS specimens obtained from 219,973 newborn samples sent to the Newborn Screening Laboratory of the Illinois Department of Public Health in Chicago [35]. Only one infant was confirmed with a positive diagnosis of MPS I and the incidence was therefore 1 in 219,793 newborns. Pseudodeficiencies for IDUA and alpha-glucosidase were detected more often than true deficiencies. The largest number of positive screening test results was for MPS I. Although many of these positive infants (58%) had normal leukocyte IDUA activity, and thus were classified as normal, pseudodeficiency of IDUA was commonly encountered. Twenty-eight infants showed low IDUA activity and normal urine GAGs, and molecular analysis confirmed a pseudodeficiency. In Mexico**,** a lysosomal NBS program was established in a cohort of 20,018 Mexican patients over the course of 3 years in a closed Mexican Health System (Petroleos Mexicanos (PEMEX) Heath Services) that covers oil company workers and their families [36]. A multiplex MS/MS enzymatic assay for six LSDs, including MPS I, Pompe disease, Fabry disease, Gaucher disease, Niemann-Pick disease type A/B, and Krabbe disease, was performed. This is the first multiplex Latin-American study of six LSDs detected through an NBS program. The authors report the final distributions included 11 Pompe disease, five Fabry disease, two MPS I, and two Niemann-Pick disease type A/B. An interesting finding emerged for the MPS I reports: two patients were found to be compound heterozygous for the c.965 T > A (p.Val322Glu) variant, one with a previously reported pathogenetic mutation c.1861C > T (p.Arg621Ter) and the other with a variant of unknown significance (VUS) c.701G > C (p.Ser234Thr) and as reported [33] in the Illinois NBS, these patients can be considered nonaffected but as pseudodeficiency.

In Brazil, a program of NBS for LSDs has been performed by a private laboratory in over 10,000 newborns, and MPS I was suspected in two babies [37]. In one, the molecular analysis of the IDUA gene identified a genotype with a possibly pathogenic variant, c.251G > C p.(Gly84Ala), and the variant, c.246C > G (p.His82Gln), associated with pseudodeficiency; the conclusion was that the baby presented pseudodeficiency for MPS I. In the second baby, the identification of a known pathogenic variant in heterozygosis c.1205G > A (p.Trp402Ter) was reported. Urine GAGs were normal in both babies.

Since November 2014 in the Tuscany and Umbria Regions of Italy, a pilot project for NBS by LC-MS/MS for Pompe disease, Fabry disease, and MPS I diseases has been conducted [38]. The enzymatic analysis was carried out on the same DBS taken at 48–72 h of life and used for expanded metabolic NBS, without the need for additional samples and with the same timing applied for babies weighing < 1800 g and babies receiving transfusions and/or parenteral nutrition. The enzyme tests were performed on voluntary basis only on DBS from infants whose parents had signed the specific informed consent. The positive infants were recalled and underwent clinical-metabolic evaluation for confirmatory diagnosis and follow-up. IDUA activity in newborn DBS was quantitated using a modified multiplex MS/MS method [19]. If the enzymatic activity on newborn DBS was below the cutoff value (normal value > 5.4 μmol/L/h) on the same newborn DBS, a single IDUA test was performed and, if the low value was confirmed, a second sample was requested. Infants with very low (< 1 μmol/L/h) IDUA activity on DBS were considered to be at high risk and all infants in this group underwent urgent clinical and biochemical analysis (enzymatic assay on leukocytes, qualitative and quantitative glycosaminoglycans assay). All infants in the high-risk group and all infants with confirmed low IDUA in the second sample were referred to the Clinical Newborn Screening Unit for clinical and biochemical evaluation. Molecular analysis of the IDUA gene was performed in all infants with confirmed low IDUA activity. Out of 64,907 newborns screened, 12 were positive for IDUA deficiency (MPS I), and eight were confirmed at retesting. IDUA gene molecular analysis showed seven infants to show either homozygosity or compound heterozygosity for pseudodeficiency alleles. The two known pseudodeficiency alleles, c.235G > A (p.Ala79Thr) and c.246C > G (p.His82Gln), were demonstrated in four and three infants, respectively, six of which were of African origin. An older brother, 10 years old, showed the same genotype and did not show signs of MPS I. Only one infant had slightly elevated urine quantitative GAGs but a normal qualitative pattern, and molecular analysis demonstrated compound heterozygosity for the pseudodeficiency allele c.246C > G (p.His82Gln) and for a stop-codon variant c.1205G > A p.(Trp402Ter) (Table 1). At recall for clinical evaluation no patients appear to be clinically affected. The follow-up of the patient with elevated urine total GAGs showed normal total GAGs in urine at 1 year, the qualitative GAGs analysis was always normal, and the patient had no physical finding suggestive of MPS I.

**Table 1:** Biochemical and mutational analysis of positive mucopolysaccharide type I (MPS I) newborns in Tuscany and Umbria Regions, Italy by lysosomal storage disease (LSD) screening

Patient no.	Gender	Ethnic origin	Country	NBS enzyme activity, triplex (N.V. > 3.74 μmol/L/h)	NBS enzyme activity, single reaction (N.V. > 5.4 μmol/L/h)	Gene	cDNA variation	Protein variation	Mutation type	Phenotype
P1-IDUA	F	European	Italy	1.36	3.4	IDUA	c.246C > G/ c.1205G > A	p.His82Gln/ p.Trp402*	Missense Nonsense	Pseudo/Hurler
P2-IDUA	M	North Africa	Morocco	0.96	4.2	IDUA	c.246C > G/ c.1757C > T	p.His82Gln/ p.Ser586Phe	Missense Missense	Pseudo/VUS
P3-IDUA	M	North Africa	Morocco	1.77	4.4	IDUA	c.1598C > G/wt	p.Pro533Arg/wt	Missense	Hurler/wt
P4-IDUA	F	European	Italy	1.4	3.8	IDUA	c.246C > G/ c.1716C > G	p.His82Gln/ p.His572Gln	Missense Missense	Pseudo/VUS
P5-IDUA	F	West Africa	Nigeria	1.39	3.48	IDUA	c.235G > A/+	p.Ala79Thr/+	Missense	Pseudo
P6-IDUA	F	West Africa	Ivory Coast	1.4	3.52	IDUA	c.235G > A/+	p.Ala79Thr/+	Missense	Pseudo
P7-IDUA	F	West Africa	Senegal	1.47	3.9	IDUA	c.235G > A/+	p.Ala79Thr/+	Missense	Pseudo
P8-IDUA	F	West Africa	Senegal	1.47	3.9	IDUA	c.235G > A/+	p.Ala79Thr/+	Missense	Pseudo

*F* female, *IDUA* alpha-l-iduronidase, *M* male, *NBS* newborn screening, *N.V.* normal value, *VUS* variant of unknown significance, *wt* wild-type

The other Italian experience on MPS I neonatal lysosomal screening has been conducted in the northeast of Italy [39].

Since September 2015, at the Division of Metabolic Inherited Disease, University Hospital of Padua, LSD newborn screening has been added to the Regional North East Italy Expanded Newborn Screening program, and 66,491 dB have been assayed for MPS I (IDUA). According to the NBS protocol, samples were collected between 36 and 48 h of life on the same card used for the other NBS tests; a second sample was required for premature babies (< 34 gestational weeks and/or weight < 2000 g) and for sick newborns (those receiving transfusions or parenteral nutrition). Determination of enzyme activities was performed by LC-MS/MS utilizing the NeoLSD kit (PerkinElmer, Turku, Finland) containing the buffer, mobile phase, substrates, and internal standards for assaying the DBS activities of acid-alpha-glucosidase (GAA), alpha-galactosidase (GLA), IDUA, and glucocerebrosidase (ABG).

To overcome false positive recall, the following cutoff system was used: if the enzymatic value was below the high-risk cutoff of 0.2 multiple of median (MOM), a second spot was requested. If the activity of the second spot was still below the cutoff, the infant was referred to our unit for confirmatory testing and clinical follow-up.

Of those 66,491 newborns screened, 24 neonates had an initial positive screening test for MPS I. Low activity was confirmed in 13, who received confirmatory testing. In these high-risk patients, physical examination, biochemical tests including heparan sulfate and dermatan sulfate in urine by MS/MS, and mutation analyses were requested (Table 2).

**Table 2:** Biochemical and mutational analysis of newborns with mucopolysaccharide type I (MPS I) deficiency in northeast Italy by lysosomal storage disease (LSD) screening

Patient no.	Sex	Ethnic origin	Country	Enzyme activity screening	Gene	cDNA variation	Protein variation	Mutation type	Phenotype	Zygosity
P1-IDUA	M	West Africa	Burkina Faso	0.1	IDUA	c.235G > A	p.Ala79Thr	Missense	Pseudo	Homozygous
P2-IDUA	F	North Africa	Morocco	0.22	IDUA	c.1598C > G	p.Pro533Arg	Missense	Hurler/Scheie	Homozygous
P3-IDUA	M	North Africa	Morocco	0.41	IDUA	c.235G > A c.1081G > A c.1743C > G	p.Ala79Thr p.Ala361Thr p.Tyr581Ter	Missense Missense Nonsense	Pseudo Pseudo Hurler	Heterozygous Heterozygous Heterozygous
P4-IDUA	M	West Africa	Niger	0.54	IDUA	c.235G > A c.296C > T c.667G > A	p.Ala79Thr p.Thr99Ile p.Asp223Asn	Missense Missense Missense	Pseudo Pseudo Pseudo	Heterozygous Heterozygous Heterozygous
P5-IDUA	F	West Africa	Gambia	0.58	IDUA	c.235G > A c.1081G > A	p.Ala79Thr p.Ala361Thr	Missense Missense	Pseudo Pseudo	Homozygous Homozygous
P6-IDUA	F	European	Italy	0.71	IDUA	c.46_57del c.246C > G	p.(Ser16_Ala19del) p.His82Gln	Deletion Missense	Hurler Pseudo	Heterozygous Heterozygous
P7-IDUA	M	West Africa	Niger	0.72	IDUA	c.235G > A	p.Ala79Thr	Missense	Pseudo	Homozygous
P8-IDUA	M	North Africa	Morocco	1.16	IDUA	c.787A > T c.1757C > T	p.Arg263Trp p.Ser586Phe	Missense Missense	Pseudo VUS	Heterozygous Heterozygous
P9-IDUA	F	North Africa	Morocco	0.53	IDUA	c.235G > A	p.Ala79Thr	Missense	Pseudo	Heterozygous
P10-IDUA	F	European	Italy	0.55	IDUA	c.1577 T > C	p.Leu526Pro	Missense	VUS	Homozygous
P11-IDUA	F	West Africa	Niger	0.59	IDUA	c.787A > T c.1949C > T	p.Arg263Trp p.Pro650Leu	Missense Missense	Pseudo VUS	Heterozygous Heterozygous
P12-IDUA	F	West Africa	Camerun	0.66	IDUA	c.235G > A	p.Ala79Thr	Missense	Pseudo	Heterozygous
P13-IDUA	F	West Africa	Niger	2.21	IDUA	c.235G > A	p.Ala79Thr	Missense	Pseudo	Heterozygous

*F* female, *IDUA* alpha-l-iduronidase, *M* male, *VUS* variant of unknown significance

Only one of 13 patients studied had elevated urine GAGs and was homozygous for an IDUA mutation encoding p.Pro553Arg (patient P2; Table 2), which was predictive of the MPS I Hurler/Scheie phenotype [40, 41]. For this patient, ERT was started at the age of 1 month.

All the other neonates showed pseudodeficiency alleles (e.g., encoding p.Ala79Thr, p.His82Gln, p.Asp223Asn). p.Ala79Thr allele is the most common pseudodeficiency reported allele in newborns of African descent as well as in the African-American population [38]. Two newborns (patients P3 and P6) were identified as carriers of a severe pathogenic mutation and a pseudodeficiency allele (Table 2).

The result of this study has shown an incidence of the MPS I disease of 1 in 66,491 newborns, with a positive predictive value (PPV) of 4.2%.

### Newborn screening programs for other MPS

In the last years, NBS techniques have been developed for several MPS [13, 26–29, 42]. Only a few pilot projects have just begun or are being organized for other types of MPS. A newborn screening for MPS I and MPS II in babies born in Osaka City University Hospital has been reported [43]. During this study, similarly to NBS in MPS I, two novel pseudodeficiency alleles, p.Pro284Leu and p.Pro260His, of the iduronate 2-sulfatase (I2S) gene have been detected. Healthy male family members with p.Pro284Leu have been reported [43].

In the US, MPS II has not been nominated for inclusion in the RUSP (Recommended Uniform Screening Panel) but, because of the availability of ERT and the possibility of multi-tests, there are plans to include it in the NBS programs. A small-scale study of NBS for MPS II has been performed [44] using fluorimetric enzyme assay among 1426 newborns screened; one was identified with reduced I2S and the DBS samples were de-identified and clinical evaluation and molecular analysis was not performed.

It is important to remember that, in an infant who has a positive MPS II NBS, I2S deficiency should have confirmatory testing performed with leukocyte I2S and arylsulfatase B or A enzyme activities. If only I2S activity is low, genetic testing for MPS II should be performed; if there is a multiple sulfatase deficiency, the SUMF1 gene should be sequenced to confirm multiple sulfatase deficiency (MSD). In the near future, a combined multiplex assay for MPS, reporting for IDUA (MPS I), I2S (MPS II), GALNS (MPS IVA), ARSB (MPS VI) [26], will allow direct assessment versus specific molecular analysis.

A newborn screening pilot study program for MPS IVA has been reported [45]. Between December 2013 and November 2015, a total of 7415 dB samples were collected from neonates born in the main medical center and three other branches of MacKay Memorial Hospital in Taiwan. Written informed consents were obtained from parents prior to the screening process. Screening involved measuring the quantity of GALNS in DBS from newborn infants using the Bio-Plex immunoassay. Eight infants whose GALNS levels were below the cutoff value were recalled for a second DBS collection, but no positive case has been found in this study [45].

### Ethical issues

Early diagnosis, early treatment, and better clinical outcome are the arguments for NBS programs (Table 3). Clinical heterogeneity, the inability to predict phenotype, and the lack of consensus about when to begin the treatment are corresponding criticisms (Table 4). A recall frequently causes anxiety and stress for the parents and, by a pilot NBS study for MPS I, we have learned that we have a number of positive screenings for pseudodeficiencies (false positive) and for variants of unknown significance (VUS). Critics of NBS also argue that is not just a panel of screening tests, but rather a multifactorial care pathway involving parental education, diagnosis, treatment, and follow-up.

**Table 3:** Mucopolysaccharidosis type I newborn screening: positive clues

• Early diagnosis → higher treatment efficacy • Early diagnosis avoids diagnostic odyssey • Disease prevalence • Prevalence of severe, mild, or subclinical phenotypes • Diagnosis in relatives • Genetic counseling

**Table 4:** Mucopolysaccharidosis newborn screening: major concerns

Enzyme activity	• No correlation genotype/phenotype • False positive in pseudodeficiency • False positive in heterozygosity
Molecular genetic analysis	• New variants of uncertain pathogenetic effect • Mild phenotypes (medicalizing, anxiety, unneeded treatment, genetic counseling)
Treatment	• Partial benefit to treatment • Late-onset/asymptomatic form

Some studies have been conducted to assess the opinions of parents of MPS patients and adult with MPS [46, 47]. A questionnaire from 249 members of MPS support groups from the US and Australia showed that 96% supported the use of NBS for MPS in situations where early treatment that favorably impacts on disease outcome is available. The most common reason supporting NBS is that NBS could avoid delay in diagnosis and the distress to patients and the family of a delayed diagnosis, with early diagnosis enabling reproductive decision-making and enabling timely initiation of treatments. These studies have identified strong support for the introduction of NBS for MPS but also important themes that are highly relevant for ethical discussion.

## Conclusions

There is a clear evidence that early intervention significantly improves the outcome of MPS I patients, and now MPS I is included in the US newborn screening panel. Other experiences are now available, including in Italy. The key factors in recommending MPS I for inclusion in NBS are the strongly improved efficacy of early-onset therapy with HSCT/ERT, ERT, and the improved performance of screening tests. Even though the MPS I newborn screening experience is still limited, it has been clearly shown that the timing of treatment impacts the outcome in infants receiving HSCT at earlier ages [40, 48–50]. They show a better cognitive outcome than those transplanted later in life.

Two studies on MPS I screening have been conducted in Italy. In the Tuscany-Umbria pilot NBS, eight infants were confirmed positive, and IDUA gene molecular analysis showed seven with either homozygosity or compound heterozygosity for pseudodeficiency alleles. p.Ala79Thr and p.His82Gln changes were demonstrated in four and three infants, respectively, six of whom were of African origin. Only one infant had transitory elevation of urine GAGs (by quantitative analysis) and she is in follow-up at the time of writing.

In the North East Italy experience, the incidence of the disorder showed one affected in 66,491 newborns screened. In this patient the treatment started at 1 month of age. Even in northeast Italy, the incidence of pseudodeficiency was very high (1:6044) with a high incidence of pseudodeficiency in patients from Africa. These data are similar, showing a high incidence of pseudodeficiency possibly related to recent immigration from Africa. On the basis of these experiences, pseudodeficiency for IDUA appears to be much more common than previously thought, representing approximately 50% of cases with a positive NBS, and this phenomenon certainly complicates the interpretation of confirmatory testing results.

**Fig. 1:**
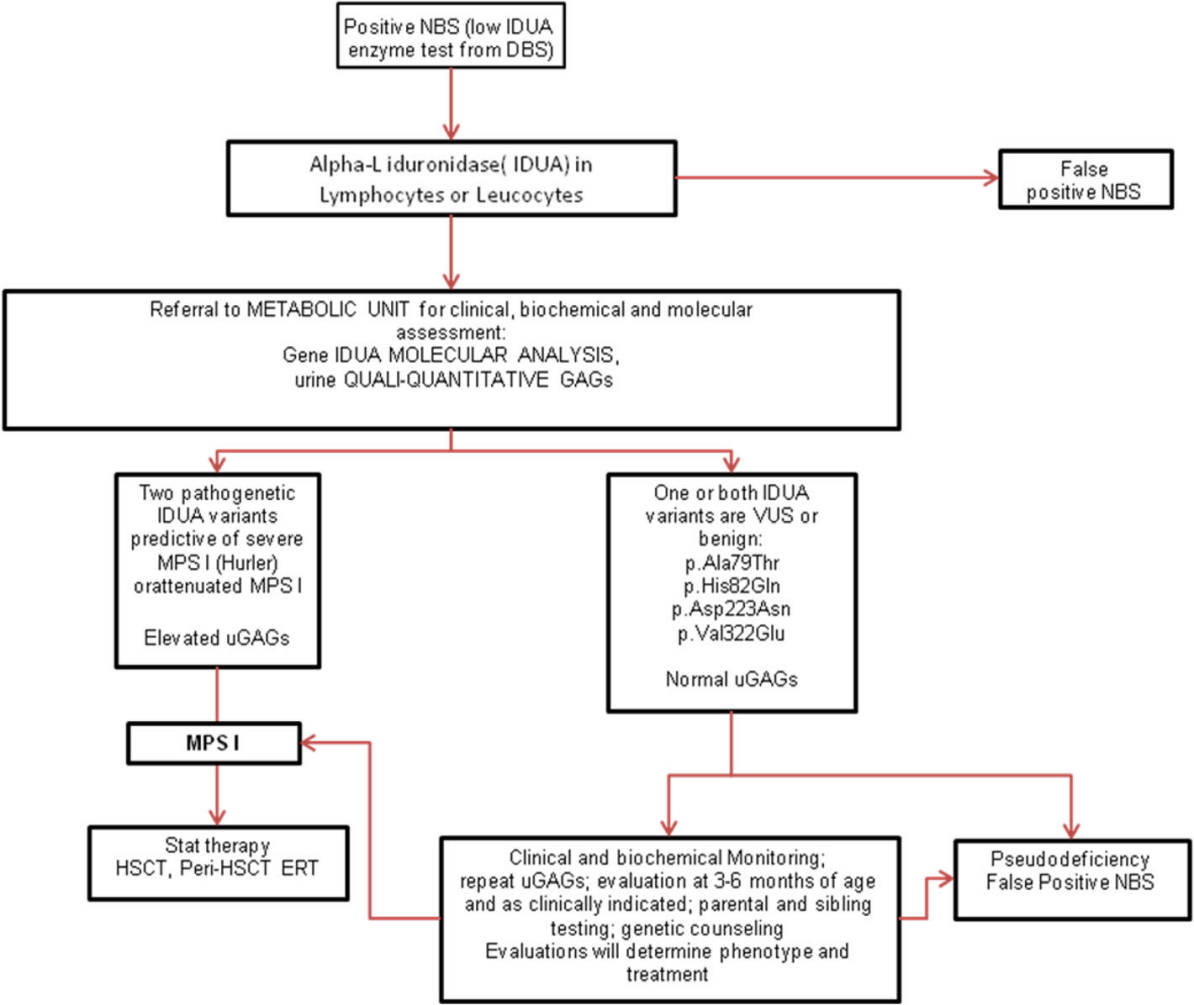
Algorithm for mucopolysaccharidosis type I (MPS I)-positive newborn screening, modified from [38]. DBS dried blood spots, ERT enzyme replacement therapy, GAG glycosaminoglycan, HSCT hematopoietic stem cell transplantation, IDUA alpha-l-iduronidase, NBS newborn screening, uGAG urinary glycosaminoglycan, VUS variant of unknown significance

On the basis of these experience, we propose an algorithm (Fig. 1) that is in agreement with what has been recently published [38].

Moreover, the increasing availability of new treatments for MPS II, IVA, VI, and VII with a better clinical outcome when started early in life and the availability of a combined multiplex assay for MPS will lead to more new pilot newborn screening programs in the near future.

## Abbreviations

ARSB: *N*-acetylgalactosamine-4-sulfatase
DBS: Dried blood spots
ERT: Enzyme replacement therapy
GAG: Glycosaminoglycan
GALNS: *N*-acetylgalactosamine-6-sulfatase
HSCT: Hematopoietic stem cell transplantation
I2S: Iduronate-2-sulfatase
IDUA: Alpha-l-iduronidase
LC-MS/MS: Liquid chromatography-tandem mass spectrometry
LSD: Lysosomal storage disease
MPS: Mucopolysaccharidosi(e)s
MS/MS: Tandem mass spectrometry
NBS: Newborn screening
VUS: Variant of unknown significance
