# Correction to: Newborn screening in mucopolysaccharidoses

**DOI:** 10.1186/s13052-019-0665-3

**Published:** 2019-06-11

**Authors:** Maria Alice Donati, Elisabetta Pasquini, Marco Spada, Giulia Polo, Alberto Burlina

**Affiliations:** 1 Metabolic and Muscular Unit, Regional Reference Centre Expanded Newborn Screening, Meyer Children Hospital, Florence, Italy; 2 Division of Inherited Metabolic Diseases, Regional Center for Expanded Neonatal Screening, Department of Women and Children’s Health, University Hospital of Padova, Via Orus 2/B, 35129 Padova, Italy; 3 Department of Pediatrics, Ospedale Regina Margherita, P.zza Polonia, 94, 10126 Torino, Italy

**Correction to: Ital J Pediatr**

**https://doi.org/10.1186/s13052-018-0552-3**

The original article [1] contains an unintended omission of a statement of competing interests. As such, the authors would like to declare the following complete statement of competing interests:
